# Trimethyl­ammonium dichlorido­triphenyl­stannate(IV)

**DOI:** 10.1107/S1600536812038457

**Published:** 2012-09-19

**Authors:** Tidiane Diop, Libasse Diop, Jerry P. Jasinski, Amanda C. Keeley

**Affiliations:** a Laboratoire de Chimie Minerale et Analytique, Département de Chimie, Faculté des Sciences et Techniques, Université Cheikh Anta Diop, Dakar, Senegal; b Department of Chemistry, Keene State College 229 Main Street Keene, NH 03435-2001, USA

## Abstract

In the structure of the title monomeric coordination salt, (C_3_H_10_N)[Sn(C_6_H_5_)_3_Cl_2_], the Sn^IV^ atom is five coordinate, with the SnC_3_Cl_2_ entity in a *trans* trigonal–bipyramidal arrangement and the chlorine atoms in apical positions. In the crystal, the cations and anions are connected by N—H⋯Cl hydrogen bonds.

## Related literature

For medical applications of tin(IV) compounds, see: Evans & Karpel (1985 ▶); Gielen (2002 ▶); Davies *et al.* (2008 ▶). For literature on organotin(IV) compounds, see: Chandrasekhar & Baskar (2003 ▶); Samuel *et al.* (2002 ▶); Nath *et al.* (2003 ▶). For related structures, see: Ng (1999 ▶, 1995 ▶); Harrison *et al.* (1978 ▶); Nayek *et al.* (2010 ▶); Sow *et al.* (2012 ▶); De Lorentiis *et al.* (2011 ▶).
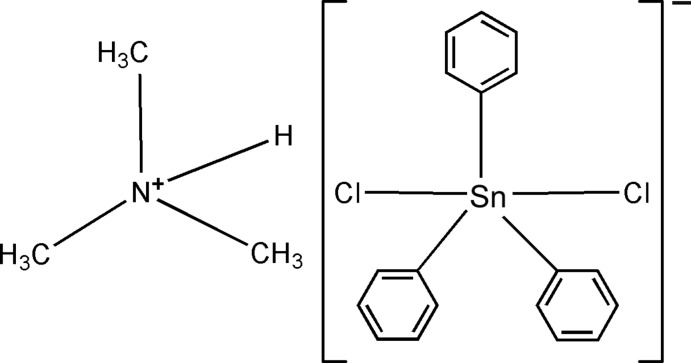


## Experimental

### 

#### Crystal data

(C_3_H_10_N)[Sn(C_6_H_5_)Cl_2_]
*M* *_r_* = 481.01
Monoclinic, 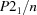

*a* = 9.2650 (2) Å
*b* = 15.6882 (4) Å
*c* = 14.7891 (3) Å
β = 90.941 (2)°
*V* = 2149.32 (8) Å^3^
*Z* = 4
Cu *K*α radiation
μ = 11.75 mm^−1^
*T* = 173 K
0.34 × 0.22 × 0.16 mm

#### Data collection

Oxford Diffraction Xcalibur Eos Gemini diffractometer
Absorption correction: multi-scan (*CrysAlis PRO* and *CrysAlis RED*; Oxford Diffraction, 2010 ▶) *T*_min_ = 0.328, *T*_max_ = 1.000
13300 measured reflections
4143 independent reflections
3739 reflections with *I* > 2σ(*I*)
*R*_int_ = 0.054

#### Refinement

*R*[*F* ^2^ > 2σ(*F* ^2^)] = 0.030
*wR*(*F* ^2^) = 0.083
*S* = 1.06
4143 reflections
229 parameters
H-atom parameters constrained
Δρ_max_ = 0.53 e Å^−3^
Δρ_min_ = −0.53 e Å^−3^

### 

Data collection: *CrysAlis PRO* (Oxford Diffraction, 2010 ▶); cell refinement: *CrysAlis PRO*; data reduction: *CrysAlis RED* (Oxford Diffraction, 2010 ▶); program(s) used to solve structure: *SHELXS97* (Sheldrick, 2008 ▶); program(s) used to refine structure: *SHELXL97* (Sheldrick, 2008 ▶); molecular graphics: *SHELXTL* (Sheldrick, 2008 ▶); software used to prepare material for publication: *SHELXTL*.

## Supplementary Material

Crystal structure: contains datablock(s) I, I-2, New_Global_Publ_Block. DOI: 10.1107/S1600536812038457/pk2438sup1.cif

Structure factors: contains datablock(s) I. DOI: 10.1107/S1600536812038457/pk2438Isup2.hkl

Additional supplementary materials:  crystallographic information; 3D view; checkCIF report

## Figures and Tables

**Table 1:** Hydrogen-bond geometry (Å, °)

*D*—H⋯*A*	*D*—H	H⋯*A*	*D*⋯*A*	*D*—H⋯*A*
N1—H1⋯Cl1^i^	0.91	2.21	3.087 (3)	161

Symmetry code: (i) 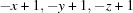.

**Fig. 1.**
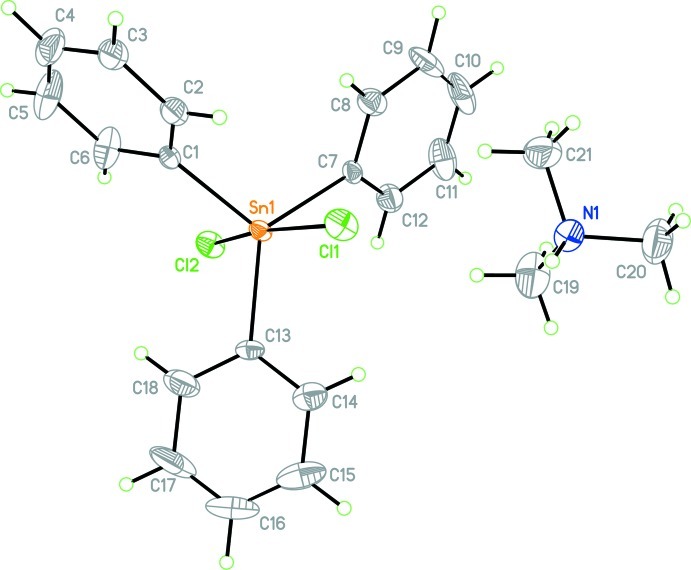
: Molecular structure of the title compound with displacement ellipsoids drawn at the 50% probability level.

**Fig. 2.**
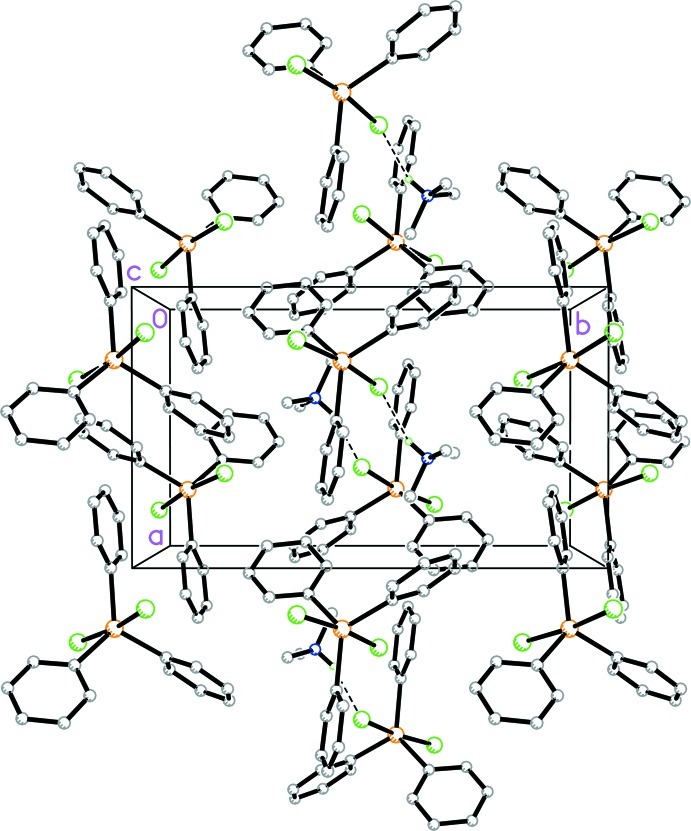
: The packing of the structure viewed along the c axis. N—H···Cl hydrogen bonds are shown as dashed lines. The remaining H atoms have been removed for clarity.

## supplementary crystallographic information

### Comment

The interest in synthesis of new organotin(IV) derivatives is related to their applications in different fields (agrochemicals, surface disinfectants and marine antifouling paints) (Evans & Karpel, 1985; Gielen, 2002; Davies *et al.*, 2008) and explains the involvement of many groups in the search for new organotin compounds (Chandrasekhar & Baskar, 2003; Samuel *et al.*, 2002; Nath *et al.*, 2003). Many compounds containing the [SnPh_3_Cl_2_]^-^ ion in the *trans* conformation have been reported (Ng, 1995, 1999; Harrison *et al.*, 1978; Nayek *et al.*, 2010; Sow *et al.*, 2012). In our search for new organotin(IV) compounds we have initiated here the study of the interactions between (CH_3_)_3_N.HCl and SnPh_3_Cl, which led to the title compound. In the [SnPh_3_Cl_2_]^-^ anion, the tin atom is located on a centre of inversion and is bonded to two Cl atoms and three phenyl groups giving a trigonal bipyramidal geometry with the chloride atoms in *trans-*positions (Fig. 1). The sum of the angles at atom Sn by the *ipso*-carbons [128.08 (12)°, 113.70 (12)°, 117.83 (12)°] is 359.61°. The corresponding axial Cl_1_—Sn—Cl_2_ angle is 171.62 (3)°, indicating a slight deviation from linearity. The Sn—C bond distances (2.135 (3) Å, 2.142 (3) Å and 2.151 (3) Å) are similar to those reported for bis(triphenylphosphanylidene)iminium dichloridotriphenylstannate(IV) (2.134 (3) Å, 2.1476 (19) Å and 2.1476 (19) Å) (De Lorentiis *et al.*, 2011). The two axial Sn—Cl distances, [Sn—Cl 2.5227 (7) Å and 2.6983 (8) Å], are very close to those reported (Sow *et al.*, 2012). The two types of Sn—Cl binding are due to disruption of NH ··· Cl hydrogen bonding on one of the chlorine atoms. The C–N–C angles of the cation are close to 109°, in agreement with the expected *sp*^3^ hybridization. The cation and the anion are connected by N—H···.Cl hydrogen bonds (Fig. 2).

### Experimental

Crystals of the title compound, [C_3_H_10_N^+^] [Sn(C_6_H_5_)_3_Cl_2_^-^], were obtained by reacting SnPh_3_Cl with (CH_3_)_3_N.HCl in ethanol in a 1/1 ratio. (CH_3_)_3_N.HCl (Merck) and SnPh_3_Cl (Aldrich) were used without further purification. The title compound was obtained by mixing in a 1/1 ratio (CH_3_)_3_N.HCl dissolved in methanol and a minimum of water and SnPh_3_Cl dissolved in methanol. The mixture was stirred for around two hours at room temperature and upon slow solvent evaporation gave prismatic crystals suitable for X-ray diffraction analysis.

### Refinement

All of the H atoms were placed in calculated positions and then refined using a riding model with C—H lengths of 0.95 Å (CH) or 0.98 Å (CH_3_) and N—H lengths of 0.90 Å (NH). The isotropic displacement parameters for these atoms were set to 1.2 (CH, NH), or 1.5 (CH_3_) times *U*_eq_ of the parent atom.

### Crystal data

**Table:**

(C_3_H_10_N)[Sn(C_6_H_5_)Cl_2_]	*F*(000) = 968
*M**_r_* = 481.01	*D*_x_ = 1.486 Mg m^−^^3^
Monoclinic, *P*2_1_/*n*	Cu *K*α radiation, λ = 1.5418 Å
Hall symbol: -P 2yn	Cell parameters from 6465 reflections
*a* = 9.2650 (2) Å	θ = 3.0–71.4°
*b* = 15.6882 (4) Å	µ = 11.75 mm^−^^1^
*c* = 14.7891 (3) Å	*T* = 173 K
β = 90.941 (2)°	Chunk, colorless
*V* = 2149.32 (8) Å^3^	0.34 × 0.22 × 0.16 mm
*Z* = 4	

### Data collection

**Table:**

Oxford Diffraction Xcalibur Eos Gemini diffractometer	4143 independent reflections
Radiation source: Enhance (Cu) X-ray Source	3739 reflections with *I* > 2σ(*I*)
Graphite monochromator	*R*_int_ = 0.054
Detector resolution: 16.15 pixels mm^-1^	θ_max_ = 71.6°, θ_min_ = 4.1°
ω scans	*h* = −11→11
Absorption correction: multi-scan (*CrysAlis PRO* and *CrysAlis RED*; Oxford Diffraction, 2010)	*k* = 0→19
*T*_min_ = 0.328, *T*_max_ = 1.000	*l* = 0→17
13300 measured reflections	

### Refinement

**Table:**

Refinement on *F*^2^	Primary atom site location: structure-invariant direct methods
Least-squares matrix: full	Secondary atom site location: difference Fourier map
*R*[*F*^2^ > 2σ(*F*^2^)] = 0.030	Hydrogen site location: inferred from neighbouring sites
*wR*(*F*^2^) = 0.083	H-atom parameters constrained
*S* = 1.06	*w* = 1/[σ^2^(*F*_o_^2^) + (0.0407*P*)^2^ + 0.8952*P*] where *P* = (*F*_o_^2^ + 2*F*_c_^2^)/3
4143 reflections	(Δ/σ)_max_ = 0.002
229 parameters	Δρ_max_ = 0.53 e Å^−^^3^
0 restraints	Δρ_min_ = −0.53 e Å^−^^3^

### Special details

**Table:**

Geometry. All e.s.d.'s (except the e.s.d. in the dihedral angle between two l.s. planes) are estimated using the full covariance matrix. The cell e.s.d.'s are taken into account individually in the estimation of e.s.d.'s in distances, angles and torsion angles; correlations between e.s.d.'s in cell parameters are only used when they are defined by crystal symmetry. An approximate (isotropic) treatment of cell e.s.d.'s is used for estimating e.s.d.'s involving l.s. planes.
Refinement. Refinement of *F*^2^ against ALL reflections. The weighted *R*-factor *wR* and goodness of fit *S* are based on *F*^2^, conventional *R*-factors *R* are based on *F*, with *F* set to zero for negative *F*^2^. The threshold expression of *F*^2^ > σ(*F*^2^) is used only for calculating *R*-factors(gt) *etc*. and is not relevant to the choice of reflections for refinement. *R*-factors based on *F*^2^ are statistically about twice as large as those based on *F*, and *R*- factors based on ALL data will be even larger.

### Fractional atomic coordinates and isotropic or equivalent isotropic displacement parameters (Å^2^)

**Table:**

	*x*	*y*	*z*	*U*_iso_*/*U*_eq_	
Sn1	0.25216 (2)	0.438102 (12)	0.741033 (13)	0.01599 (8)	
Cl1	0.34357 (9)	0.51960 (5)	0.59185 (5)	0.02688 (18)	
Cl2	0.18198 (9)	0.34254 (5)	0.87027 (5)	0.02576 (18)	
N1	0.3823 (3)	0.37067 (17)	0.38252 (19)	0.0258 (6)	
H1	0.4476	0.4132	0.3918	0.031*	
C1	0.1366 (3)	0.5479 (2)	0.7884 (2)	0.0208 (7)	
C2	0.1101 (4)	0.6179 (2)	0.7323 (2)	0.0288 (8)	
H2	0.1510	0.6199	0.6738	0.035*	
C3	0.0234 (4)	0.6853 (2)	0.7619 (3)	0.0358 (9)	
H3	0.0051	0.7324	0.7231	0.043*	
C4	−0.0352 (5)	0.6838 (2)	0.8462 (3)	0.0402 (10)	
H4	−0.0947	0.7294	0.8654	0.048*	
C5	−0.0079 (5)	0.6165 (3)	0.9030 (3)	0.0481 (12)	
H5	−0.0469	0.6159	0.9620	0.058*	
C6	0.0780 (5)	0.5481 (2)	0.8737 (3)	0.0369 (9)	
H6	0.0960	0.5014	0.9132	0.044*	
C7	0.1311 (3)	0.36151 (19)	0.64762 (19)	0.0174 (6)	
C8	0.0298 (4)	0.4005 (2)	0.5905 (2)	0.0276 (7)	
H8	0.0208	0.4608	0.5908	0.033*	
C9	−0.0578 (4)	0.3526 (3)	0.5332 (2)	0.0404 (10)	
H9	−0.1287	0.3799	0.4962	0.048*	
C10	−0.0417 (5)	0.2640 (3)	0.5299 (3)	0.0459 (11)	
H10	−0.0998	0.2309	0.4897	0.055*	
C11	0.0582 (5)	0.2257 (3)	0.5850 (3)	0.0434 (10)	
H11	0.0697	0.1655	0.5827	0.052*	
C12	0.1433 (4)	0.2732 (2)	0.6441 (2)	0.0287 (8)	
H12	0.2108	0.2451	0.6828	0.034*	
C13	0.4751 (3)	0.42610 (19)	0.7796 (2)	0.0203 (7)	
C14	0.5815 (4)	0.4029 (2)	0.7194 (3)	0.0314 (8)	
H14	0.5568	0.3940	0.6575	0.038*	
C15	0.7231 (4)	0.3926 (3)	0.7486 (3)	0.0469 (11)	
H15	0.7940	0.3747	0.7070	0.056*	
C16	0.7619 (5)	0.4079 (3)	0.8363 (4)	0.0518 (12)	
H16	0.8597	0.4017	0.8553	0.062*	
C17	0.6597 (5)	0.4321 (3)	0.8969 (4)	0.0592 (14)	
H17	0.6867	0.4428	0.9582	0.071*	
C18	0.5153 (4)	0.4413 (3)	0.8688 (3)	0.0416 (10)	
H18	0.4446	0.4581	0.9111	0.050*	
C19	0.4022 (6)	0.3087 (3)	0.4570 (3)	0.0486 (12)	
H19A	0.3296	0.2635	0.4512	0.073*	
H19B	0.3913	0.3378	0.5151	0.073*	
H19C	0.4989	0.2837	0.4540	0.073*	
C20	0.4135 (6)	0.3326 (3)	0.2933 (3)	0.0504 (11)	
H20A	0.5078	0.3043	0.2960	0.076*	
H20B	0.4149	0.3776	0.2473	0.076*	
H20C	0.3386	0.2908	0.2775	0.076*	
C21	0.2380 (4)	0.4099 (3)	0.3837 (3)	0.0465 (11)	
H21A	0.1643	0.3652	0.3835	0.070*	
H21B	0.2248	0.4460	0.3301	0.070*	
H21C	0.2290	0.4448	0.4383	0.070*	

### Atomic displacement parameters (Å^2^)

**Table:**

	*U*^11^	*U*^22^	*U*^33^	*U*^12^	*U*^13^	*U*^23^
Sn1	0.01316 (13)	0.01873 (12)	0.01599 (12)	−0.00260 (7)	−0.00193 (8)	−0.00006 (7)
Cl1	0.0250 (4)	0.0310 (4)	0.0247 (4)	−0.0069 (3)	0.0019 (3)	0.0076 (3)
Cl2	0.0272 (4)	0.0299 (4)	0.0201 (4)	−0.0032 (3)	−0.0008 (3)	0.0082 (3)
N1	0.0266 (16)	0.0215 (13)	0.0292 (15)	−0.0046 (12)	0.0047 (12)	−0.0017 (11)
C1	0.0162 (16)	0.0226 (15)	0.0236 (17)	−0.0008 (12)	−0.0050 (13)	−0.0098 (12)
C2	0.033 (2)	0.0266 (17)	0.0270 (18)	0.0065 (15)	−0.0098 (15)	−0.0050 (14)
C3	0.039 (2)	0.0297 (19)	0.038 (2)	0.0097 (16)	−0.0127 (18)	−0.0103 (15)
C4	0.038 (2)	0.0291 (19)	0.054 (3)	0.0014 (16)	0.005 (2)	−0.0202 (17)
C5	0.062 (3)	0.038 (2)	0.045 (2)	−0.007 (2)	0.030 (2)	−0.0145 (18)
C6	0.056 (3)	0.0246 (17)	0.031 (2)	−0.0022 (17)	0.0143 (18)	−0.0039 (15)
C7	0.0150 (15)	0.0246 (15)	0.0127 (14)	−0.0037 (12)	0.0012 (11)	−0.0020 (11)
C8	0.0255 (19)	0.0328 (18)	0.0243 (17)	−0.0009 (14)	−0.0011 (14)	0.0038 (14)
C9	0.027 (2)	0.069 (3)	0.0252 (19)	−0.0121 (19)	−0.0130 (15)	0.0065 (18)
C10	0.048 (3)	0.062 (3)	0.027 (2)	−0.030 (2)	−0.0060 (18)	−0.0113 (19)
C11	0.062 (3)	0.034 (2)	0.035 (2)	−0.018 (2)	−0.001 (2)	−0.0107 (17)
C12	0.033 (2)	0.0268 (17)	0.0266 (18)	0.0011 (15)	−0.0024 (15)	−0.0019 (14)
C13	0.0119 (15)	0.0214 (15)	0.0276 (18)	−0.0009 (12)	−0.0016 (13)	0.0015 (12)
C14	0.0238 (19)	0.0297 (18)	0.041 (2)	0.0000 (14)	0.0016 (16)	0.0007 (15)
C15	0.020 (2)	0.047 (3)	0.073 (3)	0.0009 (18)	0.009 (2)	0.016 (2)
C16	0.019 (2)	0.057 (3)	0.078 (4)	−0.0007 (19)	−0.014 (2)	0.020 (3)
C17	0.038 (3)	0.089 (4)	0.049 (3)	−0.009 (2)	−0.028 (2)	0.006 (2)
C18	0.024 (2)	0.065 (3)	0.036 (2)	−0.0048 (18)	−0.0083 (17)	−0.0027 (18)
C19	0.075 (4)	0.033 (2)	0.037 (2)	0.008 (2)	0.007 (2)	0.0056 (17)
C20	0.061 (3)	0.058 (3)	0.032 (2)	0.009 (2)	0.013 (2)	−0.004 (2)
C21	0.030 (2)	0.055 (3)	0.054 (3)	0.004 (2)	−0.0066 (19)	−0.016 (2)

### Geometric parameters (Å, º)

**Table:**

Sn1—C7	2.135 (3)	C10—C11	1.363 (6)
Sn1—C13	2.142 (3)	C10—H10	0.9500
Sn1—C1	2.151 (3)	C11—C12	1.385 (5)
Sn1—Cl2	2.5227 (7)	C11—H11	0.9500
Sn1—Cl1	2.6983 (8)	C12—H12	0.9500
N1—C21	1.472 (5)	C13—C18	1.385 (5)
N1—C19	1.478 (5)	C13—C14	1.388 (5)
N1—C20	1.481 (5)	C14—C15	1.384 (5)
N1—H1	0.9099	C14—H14	0.9500
C1—C6	1.381 (5)	C15—C16	1.361 (7)
C1—C2	1.396 (5)	C15—H15	0.9500
C2—C3	1.403 (5)	C16—C17	1.369 (7)
C2—H2	0.9500	C16—H16	0.9500
C3—C4	1.368 (6)	C17—C18	1.403 (6)
C3—H3	0.9500	C17—H17	0.9500
C4—C5	1.370 (6)	C18—H18	0.9500
C4—H4	0.9500	C19—H19A	0.9800
C5—C6	1.409 (5)	C19—H19B	0.9800
C5—H5	0.9500	C19—H19C	0.9800
C6—H6	0.9500	C20—H20A	0.9800
C7—C12	1.391 (5)	C20—H20B	0.9800
C7—C8	1.394 (4)	C20—H20C	0.9800
C8—C9	1.385 (5)	C21—H21A	0.9800
C8—H8	0.9500	C21—H21B	0.9800
C9—C10	1.399 (6)	C21—H21C	0.9800
C9—H9	0.9500		
			
C7—Sn1—C13	128.08 (12)	C11—C10—H10	120.3
C7—Sn1—C1	113.70 (12)	C9—C10—H10	120.3
C13—Sn1—C1	117.83 (12)	C10—C11—C12	120.9 (4)
C7—Sn1—Cl2	90.95 (8)	C10—C11—H11	119.6
C13—Sn1—Cl2	90.30 (9)	C12—C11—H11	119.6
C1—Sn1—Cl2	95.35 (10)	C11—C12—C7	120.9 (3)
C7—Sn1—Cl1	84.62 (8)	C11—C12—H12	119.5
C13—Sn1—Cl1	86.86 (9)	C7—C12—H12	119.5
C1—Sn1—Cl1	92.95 (10)	C18—C13—C14	118.3 (3)
Cl2—Sn1—Cl1	171.62 (3)	C18—C13—Sn1	118.8 (3)
C21—N1—C19	111.6 (3)	C14—C13—Sn1	122.9 (3)
C21—N1—C20	111.7 (3)	C15—C14—C13	120.6 (4)
C19—N1—C20	112.0 (3)	C15—C14—H14	119.7
C21—N1—H1	107.0	C13—C14—H14	119.7
C19—N1—H1	107.2	C16—C15—C14	120.7 (4)
C20—N1—H1	107.0	C16—C15—H15	119.6
C6—C1—C2	118.2 (3)	C14—C15—H15	119.6
C6—C1—Sn1	120.3 (3)	C15—C16—C17	119.9 (4)
C2—C1—Sn1	121.3 (3)	C15—C16—H16	120.0
C1—C2—C3	120.2 (4)	C17—C16—H16	120.0
C1—C2—H2	119.9	C16—C17—C18	120.0 (5)
C3—C2—H2	119.9	C16—C17—H17	120.0
C4—C3—C2	120.7 (4)	C18—C17—H17	120.0
C4—C3—H3	119.7	C13—C18—C17	120.4 (4)
C2—C3—H3	119.7	C13—C18—H18	119.8
C3—C4—C5	120.0 (4)	C17—C18—H18	119.8
C3—C4—H4	120.0	N1—C19—H19A	109.5
C5—C4—H4	120.0	N1—C19—H19B	109.5
C4—C5—C6	119.8 (4)	H19A—C19—H19B	109.5
C4—C5—H5	120.1	N1—C19—H19C	109.5
C6—C5—H5	120.1	H19A—C19—H19C	109.5
C1—C6—C5	121.0 (4)	H19B—C19—H19C	109.5
C1—C6—H6	119.5	N1—C20—H20A	109.5
C5—C6—H6	119.5	N1—C20—H20B	109.5
C12—C7—C8	117.9 (3)	H20A—C20—H20B	109.5
C12—C7—Sn1	122.9 (2)	N1—C20—H20C	109.5
C8—C7—Sn1	119.1 (2)	H20A—C20—H20C	109.5
C9—C8—C7	121.0 (3)	H20B—C20—H20C	109.5
C9—C8—H8	119.5	N1—C21—H21A	109.5
C7—C8—H8	119.5	N1—C21—H21B	109.5
C8—C9—C10	119.9 (4)	H21A—C21—H21B	109.5
C8—C9—H9	120.1	N1—C21—H21C	109.5
C10—C9—H9	120.1	H21A—C21—H21C	109.5
C11—C10—C9	119.3 (3)	H21B—C21—H21C	109.5
			
C7—Sn1—C1—C6	103.1 (3)	C12—C7—C8—C9	1.2 (5)
C13—Sn1—C1—C6	−83.4 (3)	Sn1—C7—C8—C9	−175.6 (3)
Cl2—Sn1—C1—C6	9.7 (3)	C7—C8—C9—C10	−2.3 (6)
Cl1—Sn1—C1—C6	−171.4 (3)	C8—C9—C10—C11	1.5 (6)
C7—Sn1—C1—C2	−72.4 (3)	C9—C10—C11—C12	0.3 (7)
C13—Sn1—C1—C2	101.0 (3)	C10—C11—C12—C7	−1.3 (6)
Cl2—Sn1—C1—C2	−165.8 (3)	C8—C7—C12—C11	0.6 (5)
Cl1—Sn1—C1—C2	13.0 (3)	Sn1—C7—C12—C11	177.3 (3)
C6—C1—C2—C3	−1.5 (5)	C7—Sn1—C13—C18	−142.3 (3)
Sn1—C1—C2—C3	174.2 (3)	C1—Sn1—C13—C18	45.3 (3)
C1—C2—C3—C4	0.7 (6)	Cl2—Sn1—C13—C18	−50.9 (3)
C2—C3—C4—C5	0.7 (6)	Cl1—Sn1—C13—C18	137.0 (3)
C3—C4—C5—C6	−1.3 (7)	C7—Sn1—C13—C14	37.0 (3)
C2—C1—C6—C5	0.9 (6)	C1—Sn1—C13—C14	−135.3 (3)
Sn1—C1—C6—C5	−174.8 (3)	Cl2—Sn1—C13—C14	128.5 (3)
C4—C5—C6—C1	0.4 (7)	Cl1—Sn1—C13—C14	−43.6 (3)
C13—Sn1—C7—C12	42.1 (3)	C18—C13—C14—C15	2.1 (5)
C1—Sn1—C7—C12	−145.3 (3)	Sn1—C13—C14—C15	−177.3 (3)
Cl2—Sn1—C7—C12	−49.0 (3)	C13—C14—C15—C16	−2.3 (6)
Cl1—Sn1—C7—C12	123.8 (3)	C14—C15—C16—C17	1.2 (7)
C13—Sn1—C7—C8	−141.3 (2)	C15—C16—C17—C18	0.0 (8)
C1—Sn1—C7—C8	31.3 (3)	C14—C13—C18—C17	−0.9 (6)
Cl2—Sn1—C7—C8	127.6 (2)	Sn1—C13—C18—C17	178.5 (3)
Cl1—Sn1—C7—C8	−59.6 (2)	C16—C17—C18—C13	−0.1 (7)

### Hydrogen-bond geometry (Å, º)

**Table:**

*D*—H···*A*	*D*—H	H···*A*	*D*···*A*	*D*—H···*A*
N1—H1···Cl1^i^	0.91	2.21	3.087 (3)	161

Symmetry code: (i) −*x*+1, −*y*+1, −*z*+1.
